# Molecular Pathways of Cardiometabolic Residual Risk in Type 2 Diabetes: Insulin Resistance, Metaflammation, and Liver–Kidney–Vascular Crosstalk

**DOI:** 10.3390/ijms27146170

**Published:** 2026-07-10

**Authors:** Antonio Maria Labate, Elena Cimino, Laura Giacomelli, Stefano Ettori, Oladayo Adigun Oladeji, Barbara Agosti

**Affiliations:** SSD Diabetologia, ASST Franciacorta, Viale Mazzini 4, 25032 Chiari, Italy

**Keywords:** type 2 diabetes, cardiometabolic risk, residual risk, insulin resistance, metaflammation, MASLD, diabetic kidney disease, endothelial dysfunction, oxidative stress, biomarkers

## Abstract

Cardiometabolic residual risk in type 2 diabetes mellitus (T2D) persists despite major advances in glucose-lowering therapy, lipid management, blood pressure control, weight reduction, and organ-protective strategies. This residual burden should not be interpreted solely as the consequence of incomplete achievement of conventional therapeutic targets, but rather as the clinical expression of persistent molecular activity involving multiple interconnected organs and pathways. Insulin resistance, metaflammation, oxidative stress, mitochondrial dysfunction, lipotoxicity, endothelial impairment, hepatic metabolic dysregulation, renal inflammation, fibrotic remodeling, and metabolic memory interact within a dynamic network linking adipose tissue, liver, kidney, immune cells, and vasculature. In this review, we discuss the biochemical and molecular drivers of cardiometabolic residual risk in T2D, with particular emphasis on impaired insulin receptor substrate/PI3K/Akt signaling, stress-kinase activation, NLRP3 inflammasome priming and assembly, MASLD-related lipotoxicity and fibrogenesis, podocyte and tubular injury, endothelial nitric oxide synthase uncoupling, AGE-RAGE signaling, and thrombo-inflammatory vascular injury. These pathways explain why biological vulnerability may persist even when conventional clinical parameters appear adequately controlled. We also examine the role of translational biomarkers and simple clinical indices, including TyG-derived indices, adiposity markers, hepatic steatosis and fibrosis scores, albuminuria, eGFR, and lipid-related markers, as accessible windows into active biological pathways. Finally, we review how contemporary therapeutic strategies may modulate selected components of this residual-risk network. A pathway-centered interpretation of T2D may support more precise residual-risk phenotyping and help move cardiometabolic care beyond isolated target control toward mechanism-based prevention. This review further links these mechanisms to the contemporary cardiovascular–kidney–metabolic (CKM) framework, as defined by the 2026 AHA/ACC/ADA/ASN CKM Guideline, and disaggregates the underlying molecular network into organ-specific pathway cascades that make the causal relationships between metabolic, inflammatory, hepatic, renal, and vascular injury more explicit.

## 1. Introduction

Type 2 diabetes mellitus (T2D) is a complex systemic disease characterized by persistent metabolic, inflammatory, vascular, renal, and hepatic abnormalities. Although traditional clinical management has long emphasized glycemic control, contemporary evidence indicates that cardiovascular, kidney, and metabolic outcomes are shaped by a broader network of interacting biological pathways [1,2,3,4,5,6,7]. The concept of cardiovascular–kidney–metabolic health provides an integrated framework for understanding how metabolic dysfunction, renal impairment, and cardiovascular injury coexist and mutually amplify each other [1].

This integrated interpretation has been further reinforced by the 2026 AHA/ACC/ADA/ASN Guideline for the Prevention, Detection, Evaluation, and Management of Cardiovascular–Kidney–Metabolic (CKM) Syndrome [8]. This guideline operationalises the organ-crosstalk concept by treating obesity, dysglycaemia, chronic kidney disease, hypertension, dyslipidaemia, and cardiovascular disease as interdependent components of a single CKM continuum rather than as isolated comorbidities. This clinical framework provides a contemporary rationale for interpreting residual risk through shared molecular pathways linking adipose tissue, liver, kidney, immune cells, and the vasculature.

Literature selection was performed as a targeted narrative review rather than as a systematic review. We searched PubMed/MEDLINE and Google Scholar, and we also screened major guideline documents and reference lists of relevant reviews and clinical trials. The search focused mainly on evidence published from 2010 to 2026, while older landmark mechanistic studies were retained when necessary to support established molecular concepts. Key terms included type 2 diabetes, cardiometabolic residual risk, cardiovascular–kidney–metabolic syndrome, insulin resistance, metaflammation, NLRP3 inflammasome, MASLD, diabetic kidney disease, endothelial dysfunction, oxidative stress, lipotoxicity, fibrosis, GLP-1 receptor agonists, SGLT2 inhibitors, finerenone, and RAAS blockade. Priority was given to mechanistic studies, translational evidence, major cardiovascular and kidney outcome trials, consensus statements, and contemporary clinical guidelines.

Despite comprehensive multifactorial pharmacological and lifestyle management, many patients with T2D continue to experience cardiovascular events, chronic kidney disease progression, heart failure, hepatic metabolic dysfunction, and microvascular complications [2,3,4,5,6,7,9,10,11]. This persistent burden is commonly described as cardiometabolic residual risk. In this review, cardiometabolic residual risk is defined as the residual biological and clinical vulnerability that persists despite partial or apparently adequate control of conventional therapeutic targets, including HbA1c, LDL cholesterol, blood pressure, body weight, albuminuria, and eGFR. This concept is related to, but broader than, classical cardiovascular residual risk. Classical cardiovascular residual risk usually refers to the persistence of atherosclerotic or cardiovascular events despite treatment of traditional risk factors, particularly LDL cholesterol. Here, we use the term in an expanded CKM sense to include persistent metabolic, hepatic, renal, vascular, inflammatory, thrombo-inflammatory, and fibrotic dimensions of risk. From a molecular perspective, residual risk is therefore not simply the amount of risk left after treatment; it is the clinical projection of biological pathways that remain active despite partial control of conventional risk factors.

A target-centered model identifies whether HbA1c, LDL cholesterol, blood pressure, body weight, eGFR, or albuminuria are above or below predefined thresholds. A pathway-centered model asks which biological process remains active and which organ axis is driving vulnerability. In T2D, persistent insulin resistance, stress-kinase activation, chronic low-grade inflammation, oxidative stress, mitochondrial dysfunction, endothelial injury, ectopic lipid accumulation, hepatic steatosis, renal inflammation, and fibrosis may continue below the surface of routine clinical measurements [9,10,11,12,13,14,15,16,17,18,19,20,21].

This persistence may also reflect metabolic and epigenetic memory. Prior exposure to hyperglycemia, lipotoxicity, and oxidative stress can leave durable molecular marks, including altered DNA methylation, histone acetylation, chromatin remodeling, and sustained transcription of pro-inflammatory or pro-fibrotic genes. These mechanisms may help explain why vascular and renal risk can persist even after later improvement in glycemic control and other clinical targets.

The liver–kidney–vascular axis is particularly relevant. MASLD is now recognized as a metabolic disease closely linked to T2D, obesity, insulin resistance, chronic kidney disease, and cardiovascular outcomes [9,10,11,22,23,24,25,26,27,28,29,30,31,32,33]. Diabetic kidney disease reflects not only renal damage but also endothelial dysfunction, inflammation, fibrosis, and systemic vascular risk [4,6,7,34,35,36,37,38,39,40,41,42,43,44,45]. Endothelial dysfunction and thrombo-inflammation represent final common pathways through which upstream metabolic and inflammatory injury becomes clinically manifest as atherosclerosis, heart failure, microvascular disease, and thrombotic events [46,47,48,49,50,51,52,53,54,55].

**Figure 1:**
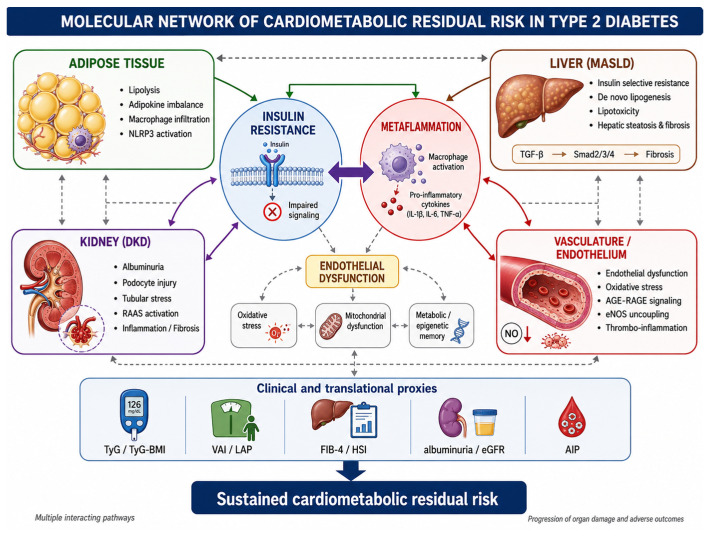
Molecular network of cardiometabolic residual risk in type 2 diabetes. Insulin resistance and metaflammation link adipose tissue dysfunction, MASLD, diabetic kidney disease, and vascular/endothelial injury with clinically accessible proxies of persistent molecular risk.

**Figure 2:**
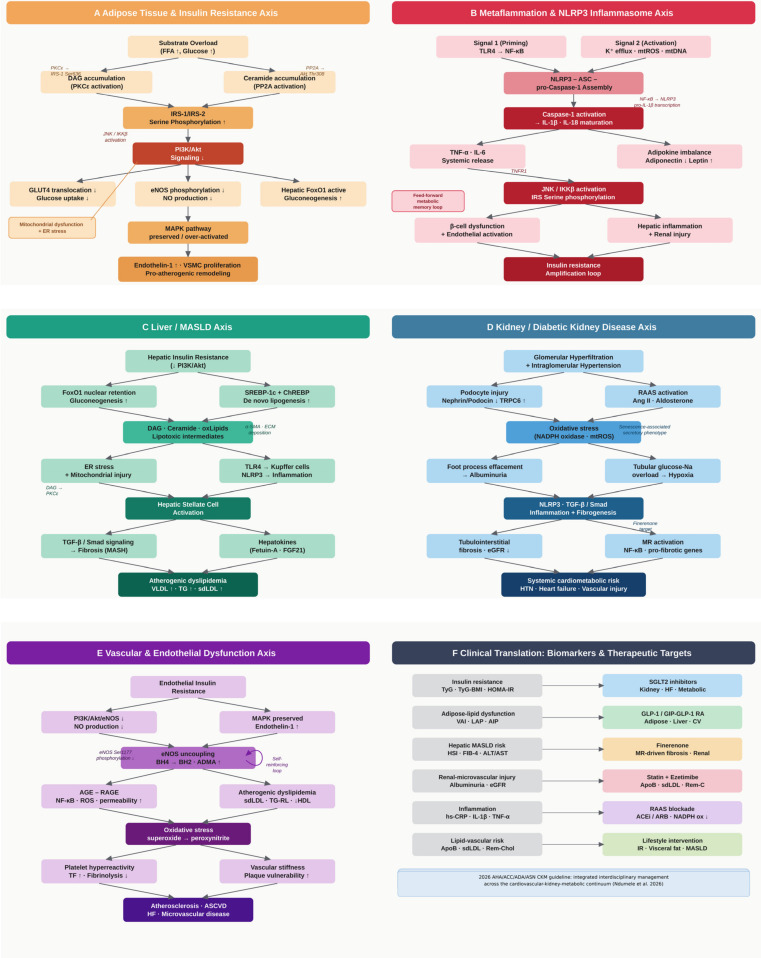
Organ-specific molecular pathway diagrams of cardiometabolic residual risk in type 2 diabetes. Each panel illustrates the main causal sequence from metabolic substrate overload and immune-metabolic activation to organ-specific injury, translational biomarkers, and therapeutic targets. Arrows indicate the direction of causal influence. (**A**) adipose tissue/insulin-resistance axis. (**B**) metaflammation/NLRP3 inflammasome axis. (**C**) liver/MASLD axis. (**D**) kidney/DKD axis. (**E**) vascular/endothelial dysfunction axis. (**F**) clinical translation, integrating biomarkers and pathway-targeted therapies. The clinical-translation panel is aligned with the 2026 AHA/ACC/ADA/ASN CKM guideline [8].

This review discusses the biochemical and molecular drivers of residual risk in T2D, focusing on insulin resistance, metaflammation, hepatic metabolic dysfunction, diabetic kidney disease, vascular injury, translational biomarkers, and pathway-oriented therapeutic modulation (Figure 1 and Figure 2).

Figure 2 further disaggregates this global network into organ-specific molecular cascades, separating the adipose/insulin-resistance, metaflammatory, liver/MASLD, kidney, vascular, and clinical-translation axes.

## 2. Insulin Resistance as the Upstream Metabolic Disturbance

Insulin resistance is one of the earliest and most pervasive molecular abnormalities in T2D. It develops across skeletal muscle, adipose tissue, liver, vascular endothelium, kidney, and pancreatic beta cells, establishing a systemic environment characterized by compensatory hyperinsulinemia, impaired glucose disposal, increased hepatic glucose production, enhanced lipolysis, ectopic lipid deposition, and reduced metabolic flexibility [12,13,14,15,16,17,18,19,20,21].

At the post-receptor level, insulin resistance is driven in part by aberrant serine/threonine phosphorylation of insulin receptor substrates, particularly IRS-1 and IRS-2. Stress-activated kinases such as c-Jun N-terminal kinase (JNK), I-kappa-B kinase β (IKKβ), and selected protein kinase C isoforms phosphorylate IRS proteins on inhibitory residues, impairing their ability to recruit the p85 regulatory subunit of PI3K and blunting downstream Akt activation [12,13,14,15,16,17,18,19,20,21]. As a consequence, glucose transport, glycogen synthesis, endothelial nitric oxide production, and anti-inflammatory insulin signaling are impaired.

Insulin resistance is characterized by a signaling imbalance rather than a global metabolic pathway failure. While PI3K/Akt-dependent metabolic signaling is impaired, mitogen-activated protein kinase-related pathways may remain relatively preserved or overactivated. This selective insulin resistance allows hyperinsulinemia to coexist with vascular smooth muscle proliferation, endothelin-1 production, inflammatory signaling, and pro-atherogenic remodeling [19,20,21,46,47,48,49,50,51,52,53,54,55].

Adipose tissue dysfunction is central to this process. When the storage capacity of adipose tissue, especially visceral adipose tissue, is exceeded, lipolysis increases, and free fatty acid flux to the liver, skeletal muscle, pancreas, kidney, and vasculature rises. This promotes ectopic lipid accumulation and generation of lipotoxic intermediates, including diacylglycerols and ceramides [17,18,19,20].

A useful way to follow this pathway is to start from substrate overload. Excess free-fatty-acid delivery and chronic hyperglycaemia promote diacylglycerol (DAG) and ceramide accumulation. DAG activates protein kinase C epsilon (PKCε) and related protein kinase C isoforms, whereas ceramides activate protein phosphatase 2A (PP2A) and interfere with Akt membrane translocation. Together with c-Jun N-terminal kinase (JNK) and I-kappa-B kinase β (IKKβ) activation, these signals favour inhibitory serine phosphorylation of IRS-1/IRS-2, thereby reducing PI3K/Akt signaling. The downstream consequences are tissue-specific: reduced GLUT4 translocation and glucose uptake in muscle, incomplete suppression of hepatic forkhead box O1 (FoxO1)-dependent gluconeogenesis, reduced endothelial nitric oxide synthase (eNOS) phosphorylation and nitric oxide production in the endothelium, persistent adipose lipolysis, and progressive beta-cell stress [12,13,14,15,16,17,18,19,20,21,46,47,48,49,50,51,52,53,54,55].

Diacylglycerols contribute to insulin resistance through tissue-specific activation of novel protein kinase C isoforms. In hepatocytes, membrane-associated diacylglycerols activate PKCε, which interferes with insulin receptor kinase activity and promotes selective hepatic insulin resistance [18]. In skeletal muscle, related lipid-mediated activation of PKC isoforms contributes to impaired IRS-1/PI3K/Akt signaling and reduced glucose uptake.

Ceramides are key mediators of lipotoxicity. They can inhibit Akt through protein phosphatase 2A-dependent dephosphorylation and through interference with Akt membrane translocation, thereby impairing insulin signaling [17,20]. Ceramides also promote mitochondrial dysfunction, oxidative stress, endoplasmic reticulum stress, apoptosis, and inflammatory activation, linking adipose dysfunction to MASLD, endothelial injury, and renal damage.

Glucotoxicity further amplifies metabolic injury. Chronic hyperglycemia increases mitochondrial reactive oxygen species generation, advanced glycation end-product formation, protein kinase C activation, polyol pathway flux, and hexosamine pathway activity [48,54,55]. These mechanisms damage endothelial cells, podocytes, tubular cells, hepatocytes, and beta cells, thereby translating metabolic excess into multi-organ injury.

Mitochondrial dysfunction contributes to both insulin resistance and residual risk. Reduced oxidative capacity, impaired mitophagy, abnormal mitochondrial dynamics, and excessive reactive oxygen species generation compromise cellular energy handling and activate inflammatory pathways [15,16]. Endoplasmic reticulum stress adds a parallel stress-response pathway in which nutrient overload and lipid accumulation activate maladaptive unfolded protein responses, JNK signaling, beta-cell dysfunction, and hepatocyte injury [12,13,14,15,16,17,18,19,20,21].

Thus, insulin resistance is not only the metabolic defect that precedes hyperglycemia. It is the upstream molecular platform from which hepatic steatosis, metaflammation, endothelial dysfunction, kidney injury, oxidative stress, and cardiometabolic residual risk develop.

## 3. Metaflammation and Immune-Metabolic Activation

Chronic low-grade inflammation, commonly described as metaflammation, is a defining molecular feature of obesity-related insulin resistance and T2D. Unlike acute inflammation, metaflammation is persistent, moderate in intensity, and directly coupled to nutrient excess, adipose tissue dysfunction, mitochondrial stress, and altered innate immune signaling [56,57,58,59,60,61,62,63,64,65].

Visceral adipose tissue is a major site where metabolic stress becomes immune activation. Adipocyte hypertrophy induces hypoxia, mechanical stress, extracellular matrix remodeling, mitochondrial dysfunction, and local cell death. These changes increase lipolysis and promote secretion of chemokines, saturated fatty acids, extracellular vesicles, and pro-inflammatory cytokines, converting adipose tissue from an energy-storage compartment into an active immune-metabolic organ [60,64,65].

Macrophage infiltration is central to adipose tissue metaflammation. In obesity and T2D, adipocyte stress and death promote recruitment of monocyte-derived macrophages and formation of crown-like structures. These macrophages acquire a predominantly pro-inflammatory phenotype and release TNF-α, IL-1β, IL-6, monocyte chemoattractant protein-1, and other mediators that impair insulin signaling locally and systemically [58,59,63,64,65].

The NLRP3 inflammasome is a key molecular platform linking metabolic danger signals to inflammatory activation. Its activation generally requires two signals. The priming signal, frequently mediated by TLR4 and NF-kappaB activation, increases transcription of NLRP3 and pro-IL-1β. The activation signal is triggered by events such as potassium efflux, lysosomal destabilization, mitochondrial dysfunction, reactive oxygen species generation, and release of mitochondrial DNA. These events promote assembly of the NLRP3-ASC-pro-caspase-1 complex, caspase-1 activation, and maturation of IL-1β and IL-18 [56,57,58,59,60,61,62].

This two-signal model is clinically important because it explains how sterile metabolic stress becomes persistent inflammation. Signal 1, usually mediated by TLR4 and NF-kappaB, primes the system by increasing transcription of NLRP3 and pro-IL-1β. Signal 2, generated by potassium efflux, lysosomal destabilization, mitochondrial reactive oxygen species, and mitochondrial DNA release, promotes assembly of the NLRP3-apoptosis-associated speck-like protein containing a CARD (ASC)-pro-caspase-1 complex. Caspase-1 then drives maturation of IL-1β and IL-18. These cytokines amplify JNK and IKKβ activity, worsen IRS serine phosphorylation, promote beta-cell dysfunction and endothelial activation, and generate a feed-forward loop in which metabolic danger signals maintain inflammatory memory even when routine clinical parameters improve [56,57,58,59,60,61,62,63,64,65].

Cytokine signaling directly interferes with insulin action. Binding of TNF-α to TNFR1 activates downstream JNK and IKKβ signaling, which phosphorylate IRS proteins on inhibitory serine residues and disrupt insulin metabolic signaling. IL-1β contributes to beta-cell dysfunction, endothelial activation, and tissue inflammation, whereas chronic IL-6 pathway activation is associated with hepatic inflammation, insulin resistance, and cardiovascular risk [56,57,58,59,60,61,62,63].

Adipokine imbalance further contributes to residual risk. Reduced adiponectin removes an important anti-inflammatory, insulin-sensitizing, anti-atherogenic, and hepatoprotective signal. Conversely, hyperleptinemia and leptin resistance may promote sympathetic activation, oxidative stress, inflammation, vascular remodeling, and renal injury. Resistin, visfatin, retinol-binding protein 4, and adipose-derived extracellular vesicles may also participate in immune-metabolic crosstalk [61,64,65].

Oxidative stress and metaflammation are mutually reinforcing. Mitochondrial reactive oxygen species, NADPH oxidase activation, uncoupled nitric oxide synthase, and AGE-RAGE signaling activate inflammatory transcription factors such as NF-kappaB and perpetuate cytokine production [47,48,54,55,62]. Inflammatory cytokines in turn increase oxidative stress and mitochondrial dysfunction, creating a feed-forward loop linking adipose tissue, liver, kidney, and vasculature.

Metaflammation also promotes a prothrombotic and pro-atherogenic phenotype. Endothelial activation, platelet hyperreactivity, increased tissue factor expression, altered fibrinolysis, and inflammatory leukocyte recruitment contribute to thrombo-inflammatory residual risk [46,47,48,49,50,51]. Thus, metaflammation is not an accessory phenomenon in T2D; it is a central biological amplifier that transforms nutrient excess into persistent molecular risk.

## 4. Liver Axis: MASLD as a Biochemical Amplifier of Residual Risk

The liver is a central metabolic hub in T2D and a major amplifier of cardiometabolic residual risk. MASLD is highly prevalent in people with T2D and reflects the hepatic expression of insulin resistance, adipose tissue dysfunction, altered lipid trafficking, mitochondrial stress, inflammation, and fibrotic remodeling [9,10,11,22,23,24,25,26,27,28,29,30,31,32,33]. The transition to MASLD nomenclature emphasizes the metabolic basis of the disease and its relationship with obesity, insulin resistance, chronic kidney disease, and cardiovascular outcomes [9,10,11].

Selective hepatic insulin resistance is a defining molecular feature of T2D. Impaired Akt signaling fails to induce nuclear exclusion and transcriptional suppression of FoxO1, leaving gluconeogenic gene expression inadequately inhibited. At the same time, nutrient-driven and hyperinsulinemia-associated activation of SREBP-1c and ChREBP promotes de novo lipogenesis [19,20,21,30,31,32,33]. This paradox explains how increased hepatic glucose production coexists with enhanced lipogenesis.

The paradox of selective hepatic insulin resistance is particularly relevant to residual risk. Reduced Akt activity fails to exclude FoxO1 from the nucleus, allowing continued transcription of gluconeogenic genes despite hyperinsulinemia. At the same time, nutrient excess and compensatory hyperinsulinemia maintain sterol regulatory element-binding protein 1c (SREBP-1c) and carbohydrate-responsive element-binding protein (ChREBP) activity, thereby increasing de novo lipogenesis. This dissociation explains why hepatic glucose overproduction and lipid synthesis coexist in T2D. The resulting triglyceride accumulation, VLDL secretion, DAG/ceramide generation, mitochondrial stress, and Kupffer-cell activation connect hepatic insulin resistance to atherogenic dyslipidaemia, systemic inflammation, and fibrogenesis [19,20,21,26,27,30,31,32,33].

De novo lipogenesis increases hepatic triglyceride accumulation and very-low-density lipoprotein secretion. SREBP-1c activation favors fatty acid synthesis, whereas ChREBP links carbohydrate flux to lipogenic gene expression. In T2D, these transcriptional programs contribute to hypertriglyceridemia, remnant lipoprotein burden, atherogenic dyslipidemia, and ectopic lipid deposition [26,27,30,31,32,33].

The severity of MASLD is determined less by neutral triglyceride storage than by lipotoxicity. Reactive lipid species, including ceramides, diacylglycerols, lysophosphatidylcholines, free cholesterol, and oxidized lipids, impair insulin signaling, damage mitochondria, activate endoplasmic reticulum stress, and trigger inflammatory and apoptotic pathways [17,18,19,20,26,27]. Ceramide-mediated inhibition of Akt and lipid-induced mitochondrial injury provide direct biochemical links between hepatic steatosis and systemic insulin resistance.

Mitochondrial dysfunction is central to MASLD progression. Excess fatty acid oxidation, electron transport chain stress, impaired mitochondrial dynamics, and increased reactive oxygen species promote hepatocyte injury. Oxidative stress activates stress kinases, inflammatory pathways, and cell death mechanisms, while also promoting hepatic stellate cell activation and extracellular matrix deposition [15,16,26,27,31].

Inflammation in MASLD involves Kupffer cell activation, monocyte-derived macrophage recruitment, inflammasome signaling, cytokine release, hepatocyte ballooning, and cell death. Lipotoxic hepatocytes release danger-associated molecular patterns that activate immune cells and hepatic stellate cells. Saturated fatty acids and free cholesterol may amplify TLR4-related signaling, reinforcing inflammatory and fibrogenic responses [26,27,31,56,57,58,59,60,61,62].

Fibrogenesis is mediated by activation of hepatic stellate cells and profibrotic pathways, particularly transforming growth factor-beta/Smad signaling. Activated stellate cells acquire a myofibroblast-like phenotype, increase α-smooth muscle actin expression, and deposit extracellular matrix proteins. Fibrosis is the strongest histological determinant of liver-related outcomes and also identifies a phenotype of advanced systemic metabolic dysfunction [22,24,27,66,67].

MASLD is associated with cardiovascular disease through mechanisms that extend beyond shared risk factors. Hepatic insulin resistance, atherogenic dyslipidemia, systemic inflammation, oxidative stress, endothelial dysfunction, altered coagulation, and hepatokine secretion contribute to vascular injury [23,24,25,26,28,30,31,32,33]. Hepatokines such as fetuin-A, FGF21, and selenoprotein P illustrate how the liver functions as an endocrine-metabolic organ capable of influencing insulin sensitivity, vascular function, inflammation, and energy metabolism [32,33].

Thus, MASLD is not a passive comorbidity in T2D. It represents a biochemical amplifier of residual risk through the integration of insulin resistance, lipotoxicity, inflammation, fibrosis, dyslipidemia, hepatokine signaling, and vascular injury.

## 5. Kidney Axis: Renal Dysfunction, Albuminuria, and Vascular Injury

Diabetic kidney disease is a major organ manifestation of cardiometabolic residual risk in T2D. It should not be interpreted only as a downstream microvascular complication of chronic hyperglycemia, but as an active biological node in which metabolic stress, glomerular hemodynamic changes, tubular dysfunction, endothelial injury, inflammation, oxidative stress, and fibrosis converge [4,6,7,34,35,36,37,38,39,40,41,42,43,44,45].

Albuminuria serves as a pivotal translational proxy because it reflects glomerular endothelial dysfunction, podocyte injury, basement membrane alterations, tubular stress, and inflammatory activation. Its association with cardiovascular events indicates that albuminuria captures a systemic microvascular phenotype rather than isolated renal leakage [4,6,7,34,35,36,37,38,39,40].

Podocyte injury is an early and central component of diabetic kidney disease. Glomerular hyperfiltration-induced mechanical stretch, local angiotensin II upregulation, oxidative stress, insulin resistance, and inflammatory mediators disrupt the podocyte actin cytoskeleton and slit diaphragm integrity. Downregulation or altered organization of nephrin and podocin, activation of TRPC6-related calcium influx, and loss of podocyte adhesion molecules contribute to foot process effacement, albuminuria, and glomerulosclerosis [34,35,36,40].

Proximal tubular epithelial cells are active immunometabolic hubs under high sodium-glucose reabsorption workloads. Increased filtered glucose and sodium load enhances ATP demand and oxygen consumption, promoting tubular hypoxia, mitochondrial stress, oxidative injury, and inflammatory signaling. Chronic metabolic strain can induce a senescence-associated secretory phenotype, with secretion of profibrotic and pro-inflammatory mediators including TGF-β1 [34,35,36].

RAAS activation contributes to both hemodynamic and molecular injury. Angiotensin II promotes efferent arteriolar vasoconstriction, glomerular hypertension, NADPH oxidase activation, oxidative stress, endothelial dysfunction, podocyte injury, and fibrosis. Aldosterone and mineralocorticoid receptor activation further induce inflammatory and profibrotic transcriptional programs in renal and vascular cells [38,39,42].

Inflammation and fibrosis are key determinants of residual renal risk. Macrophage infiltration, inflammasome activation, cytokine release, TGF-β/Smad signaling, epithelial-to-mesenchymal transition-like responses, and extracellular matrix deposition contribute to progressive tubulointerstitial fibrosis [34,35,36]. These pathways may remain active even after improvement in glycemic control, supporting the relevance of metabolic memory and persistent tissue remodeling.

The causal pathway from renal metabolic stress to systemic risk can therefore be summarised as follows. Glomerular hyperfiltration and RAAS activation increase intraglomerular pressure and oxidative stress; podocyte stretch and angiotensin II signaling disrupt nephrin, podocin, transient receptor potential cation channel C6 (TRPC6)-related calcium handling, and slit-diaphragm integrity, leading to albuminuria. In parallel, proximal tubular glucose-sodium overload increases oxygen demand and promotes hypoxia, mitochondrial stress, senescence-associated secretory signalling, and inflammatory activation. NLRP3 activation, mineralocorticoid receptor signalling, and TGF-β/Smad pathways then favour extracellular-matrix deposition and tubulointerstitial fibrosis, linking DKD progression to hypertension, heart failure, vascular stiffness, and systemic cardiometabolic risk [34,35,36,37,38,39,40,41,42,43,44,45].

The kidney also participates in systemic cardiometabolic regulation. Altered sodium-glucose handling, glomerular hyperfiltration, intrarenal hypoxia, neurohormonal activation, impaired natriuresis, and reduced kidney function contribute to hypertension, volume expansion, heart failure risk, vascular stiffness, and systemic inflammation [1,6,7,37,38,39,40,41,42,43,44,45]. This bidirectional relationship supports the concept of a kidney–heart–metabolic axis.

SGLT2 inhibitors have clinically validated the kidney axis. Their effects include restoration of tubuloglomerular feedback, reduction in intraglomerular pressure, natriuresis, improved renal oxygen handling, metabolic substrate shifts, reduced oxidative stress, and modulation of inflammatory pathways [37,43,44,45,68,69,70,71]. Finerenone further highlights the role of inflammatory-fibrotic residual risk by antagonizing mineralocorticoid receptor-driven transcriptional activation in renal and vascular tissues [38,39,42].

Therefore, kidney injury in T2D is not merely a late complication. It is an active amplifier of cardiometabolic risk, linking albuminuria, endothelial dysfunction, podocyte and tubular injury, inflammation, fibrosis, heart failure, and vascular disease within a single residual-risk network.

## 6. Vascular Axis: Endothelial Dysfunction and Atherothrombotic Risk

The vascular endothelium is a major target and mediator of molecular injury in T2D. Endothelial dysfunction represents a central mechanism through which upstream metabolic abnormalities become clinically manifest as atherosclerosis, microvascular disease, heart failure, peripheral artery disease, and thrombotic events [46,47,48,49,50,51,52,53,54,55].

Under physiological conditions, insulin activates PI3K/Akt-dependent endothelial nitric oxide synthase (eNOS) signaling, increasing nitric oxide production and promoting vasodilation. In insulin-resistant endothelial cells, impaired Akt activation reduces phosphorylation of eNOS at Ser1177, decreasing nitric oxide generation and impairing vasoprotective signaling [19,20,21,46,49,53].

Selective endothelial insulin resistance creates a pro-atherogenic imbalance. While PI3K/Akt/eNOS signaling is attenuated, MAPK-related pathways may remain relatively preserved, promoting endothelin-1 production, vascular smooth muscle cell proliferation, inflammatory gene expression, and vascular remodeling [19,20,21,53]. This imbalance converts insulin from a predominantly vasoprotective signal into a context in which hyperinsulinemia may coexist with vascular injury.

Oxidative stress is a major driver of endothelial dysfunction. Reactive oxygen species reduce nitric oxide bioavailability, oxidize lipoproteins, damage cellular macromolecules, activate inflammatory transcription factors, and increase endothelial permeability [47,48,54,55]. Mitochondrial electron leakage, NADPH oxidase isoforms, xanthine oxidase, AGE-RAGE signaling, and uncoupled eNOS all contribute to the expansion of the intracellular reactive oxygen species pool.

eNOS uncoupling is a key biochemical event. Oxidation of tetrahydrobiopterin (BH4) to dihydrobiopterin (BH2), depletion of L-arginine, and accumulation of asymmetric dimethylarginine can shift eNOS from nitric oxide production toward superoxide generation. This creates a vicious cycle in which endothelial nitric oxide deficiency and oxidative stress amplify each other [46,47,48,49,50,51,52,53,54,55].

These biochemical changes create a self-reinforcing endothelial loop. Impaired PI3K/Akt signaling reduces eNOS Ser1177 phosphorylation and nitric oxide production, whereas tetrahydrobiopterin (BH4) oxidation to dihydrobiopterin (BH2), L-arginine depletion, and asymmetric dimethylarginine accumulation shift eNOS toward superoxide generation. Superoxide reacts with residual nitric oxide to form peroxynitrite, further oxidizing BH4 and reducing nitric oxide bioavailability. In parallel, AGE-RAGE signaling and atherogenic dyslipidaemia activate NF-kappaB, increase endothelial permeability, favour platelet hyperreactivity, and promote plaque vulnerability. This explains how metabolic injury is converted into atherothrombosis, heart failure, and microvascular disease [46,47,48,49,50,51,52,53,54,55].

The AGE-RAGE axis links chronic metabolic stress to vascular injury. Advanced glycation end-products accumulate as a consequence of hyperglycemia, oxidative stress, and aging. Their interaction with RAGE activates NF-kappaB, increases cytokine production, enhances oxidative stress, promotes endothelial permeability, and contributes to vascular stiffness and plaque vulnerability [50,54,55]. AGE-RAGE signaling also interacts with renal injury and inflammation, reinforcing multi-organ residual risk.

Atherogenic dyslipidemia further contributes to vascular residual risk. Insulin resistance is associated with increased triglyceride-rich lipoproteins, remnant cholesterol, small dense LDL particles, and low HDL cholesterol. These abnormalities promote endothelial activation, foam-cell formation, oxidative modification of lipoproteins, and plaque progression. Even when LDL cholesterol is reduced, residual lipid-related risk may persist through triglyceride-rich particles and inflammatory vascular activation [2,46,47,48,49,50,51,52,53,54,55].

Thrombo-inflammation represents another relevant dimension. T2D is associated with platelet hyperreactivity, increased tissue factor expression, enhanced coagulation activation, impaired fibrinolysis, endothelial activation, and inflammatory leukocyte recruitment [51]. Microvascular dysfunction, including capillary rarefaction, glycocalyx damage, impaired vasodilatory reserve, basement membrane thickening, and tissue hypoxia, affects kidney, retina, myocardium, peripheral nerves, skeletal muscle, and adipose tissue [52,53].

Thus, the vascular axis is the clinical translation site of residual molecular risk. It integrates insulin resistance, oxidative stress, AGE-RAGE activation, metaflammation, atherogenic dyslipidemia, kidney injury, hepatic dysfunction, eNOS uncoupling, and thrombo-inflammatory signaling into cardiovascular and microvascular events.

## 7. Translational Biomarkers and Clinical Indices of Molecular Residual Risk

The molecular network underlying cardiometabolic residual risk is complex, dynamic, and only partially measurable in routine clinical practice. Clinically accessible biomarkers and composite indices may help translate this complexity into practical risk stratification. Their role is not to replace molecular profiling but to function as low-cost proxies of insulin resistance, visceral adiposity, hepatic dysfunction, lipid-related vascular stress, renal-microvascular injury, and inflammatory burden [66,67,72,73,74,75,76,77,78,79,80,81,82,83].

The triglyceride-glucose (TyG) index is a surrogate marker of insulin resistance that integrates fasting glucose and triglycerides, two biochemical consequences of impaired insulin action [72,73,74,75,81]. Mechanistically, an elevated TyG index may reflect hepatic glucose overproduction, increased very-low-density lipoprotein secretion, adipose tissue lipolysis, impaired skeletal muscle glucose uptake, and saturated peripheral lipid-storage capacity.

TyG-derived indices, including TyG-BMI and TyG-waist circumference, integrate insulin resistance with body size or fat distribution. This is biologically relevant because visceral adiposity increases free fatty acid flux, promotes lipotoxicity, activates inflammatory pathways, and contributes to hepatic steatosis and endothelial dysfunction [17,18,19,20,21,56,57,58,59,60,61,62,63,64,65,76,77]. These indices may therefore approximate the combined burden of metabolic stress and adipose tissue dysfunction.

VAI and LAP provide additional windows into adipose-lipid dysfunction [76,77,82,83]. VAI combines anthropometric and lipid variables and may reflect altered adipose tissue secretory function, including the shift from adiponectin-dominant protective signaling toward leptin resistance, resistin-associated inflammation, and triglyceride-rich lipoprotein excess. LAP captures lipid overaccumulation and may represent abdominal lipid storage exceeding metabolically safe capacity.

AIP, based on the logarithmic triglyceride-to-HDL cholesterol ratio, reflects the balance between triglyceride-rich lipoproteins and HDL-related protective pathways [80]. In insulin-resistant states, increased triglycerides, low HDL cholesterol, remnant particles, and small dense LDL contribute to endothelial activation, oxidative stress, and plaque progression. AIP can therefore be interpreted as a proxy of lipid-driven vascular residual risk.

Hepatic indices are relevant because MASLD is a key amplifier of systemic risk. Hepatic steatosis scores identify probable liver fat accumulation, whereas FIB-4 estimates fibrosis-related risk [66,67,78,79]. These tools are imperfect and cannot replace dedicated liver assessment, but they may identify patients in whom hepatic lipotoxicity, inflammation, and fibrotic remodeling contribute to residual vulnerability.

Renal biomarkers complete this translational profile. eGFR reflects filtration capacity, whereas albuminuria captures glomerular endothelial injury, podocyte dysfunction, tubular stress, and systemic microvascular damage [4,6,7,34,35,36,37,38,39,40,41,42,43,44,45]. Their integration with insulin-resistance, adiposity, lipid, and hepatic indices may help identify liver–kidney–vascular phenotypes of residual risk.

The primary intrinsic limitation of these indices relies on their indirect nature. They do not measure intracellular kinase activation, inflammasome assembly, eNOS coupling, hepatocyte lipotoxicity, or podocyte stress. Their value lies in providing accessible clinical windows into active biological pathways, especially when advanced molecular profiling is not available.

The main molecular pathways, clinical proxies, and potential therapeutic modulators are summarized in Table 1.

## 8. Therapeutic Modulation of Molecular Residual Risk

Therapeutic strategies in T2D increasingly extend beyond glucose lowering. A pathway-oriented approach interprets treatments according to their capacity to modulate insulin resistance, adipose tissue dysfunction, hepatic steatosis, renal hemodynamic stress, oxidative injury, inflammation, endothelial dysfunction, and fibrotic remodeling [2,3,4,5,6,7,68,69,70,71,84,85,86,87,88,89,90,91].

In line with the 2026 AHA/ACC/ADA/ASN CKM guideline, therapeutic modulation should not be interpreted as sequential correction of isolated targets, but as coordinated, risk-based treatment intensification across the CKM continuum [8]. This approach requires simultaneous attention to lifestyle and adiposity, blood pressure, apoB-containing lipoproteins, renal protection, glucose-lowering strategies with proven cardiovascular and kidney benefit, and anti-fibrotic or anti-inflammatory mechanisms when clinically indicated. In this perspective, SGLT2 inhibitors, GLP-1 receptor agonists, dual GIP/GLP-1 receptor agonism, RAAS blockade, finerenone, and lipid-lowering therapy are complementary pathway modulators rather than competing single-target interventions.

The strength of evidence is not identical across all pathways discussed in this section. For several therapeutic classes, including SGLT2 inhibitors, GLP-1 receptor agonists, finerenone, RAAS blockade, and lipid-lowering therapy, cardiovascular or kidney outcome evidence is established in clinical trials. By contrast, proposed mechanisms involving AMPK, SIRT1, PGC-1α, sodium-hydrogen exchanger modulation, inflammasome activity, hepatokines, and selected endothelial pathways should be interpreted mainly as experimental or translational explanations that may help account for clinical benefit, rather than as independently proven clinical endpoints in every setting. Accordingly, the mechanistic interpretation below is intended to connect biological plausibility with clinical evidence without implying that all molecular pathways have equivalent levels of human validation.

Lifestyle intervention remains the foundational modulator of molecular residual risk. Weight loss, physical activity, nutritional quality, smoking cessation, and improved cardiorespiratory fitness can improve insulin sensitivity, reduce visceral adiposity, decrease hepatic fat, modulate inflammatory signaling, improve mitochondrial function, and enhance endothelial nitric oxide bioavailability. These effects are biologically broad and cannot be reduced to HbA1c lowering alone.

Lipid-lowering therapy reduces a dominant causal pathway of atherosclerotic risk by lowering apoB-containing lipoproteins. However, triglyceride-rich lipoproteins, remnant cholesterol, vascular inflammation, oxidative stress, and thrombo-inflammatory signaling may remain active despite LDL cholesterol reduction [2,46,47,48,49,50,51,52,53,54,55]. This explains why lipid lowering is essential but not sufficient to silence the entire residual-risk network.

RAAS blockade reduces intraglomerular pressure, albuminuria, oxidative stress, inflammation, and fibrosis [4,6,7,34,35,36,37,38,39,40,41,42,43,44,45]. At the molecular level, inhibition of angiotensin II signaling reduces NADPH oxidase activation, endothelial injury, podocyte stress, and profibrotic TGF-β-related signaling, thereby affecting both the renal and vascular components of residual risk.

SGLT2 inhibitors exemplify mechanism-targeted therapy. Beyond glycosuria, they restore tubuloglomerular feedback, reduce intraglomerular hypertension, decrease tubular workload, promote natriuresis, improve renal oxygen handling, and alter cellular energetics [37,43,44,45,68,69,70,71]. Several mechanistic models suggest activation of fasting-like pathways involving AMPK, SIRT1, and PGC-1α, with improved mitochondrial efficiency, reduced oxidative stress, and attenuation of inflammatory signaling. In the myocardium and vasculature, modulation of sodium-hydrogen exchanger activity and improved ionic homeostasis may further contribute to protection.

GLP-1 receptor agonists modulate multiple components of residual risk through receptor-mediated cAMP generation, protein kinase A activation, and signaling via the exchange protein directly activated by cAMP [84,85,89,90,91]. These pathways improve insulin secretion in a glucose-dependent manner and may also reduce appetite, body weight, inflammation, endothelial dysfunction, and atherosclerotic processes. Anti-inflammatory effects may involve attenuation of NF-kappaB signaling, reduced macrophage activation, improved adipose tissue biology, and indirect reduction in hepatic steatosis through weight loss and improved insulin sensitivity.

Dual GIP/GLP-1 receptor agonism may expand this paradigm by simultaneously targeting nutrient sensing, adipose tissue buffering capacity, insulin secretion, weight regulation, and hepatic metabolic stress [28,86,87,88]. Tirzepatide improves glycemic control and body weight and has shown promising effects in MASH with fibrosis, supporting the concept that incretin-based therapies can modulate adipose–liver crosstalk and hepatic lipotoxicity [28].

Mineralocorticoid receptor antagonism with finerenone targets inflammatory and fibrotic residual risk. Finerenone acts as a non-steroidal mineralocorticoid receptor antagonist that prevents recruitment of transcriptional co-activators required for expression of pro-inflammatory and profibrotic genes in renal and vascular cells [38,39,42]. This mechanism is relevant to albuminuria, tubulointerstitial fibrosis, vascular inflammation, and cardiovascular risk.

Anti-inflammatory strategies remain an evolving area. The success of inflammation-targeted interventions in atherosclerosis has reinforced the biological relevance of residual inflammatory risk, but routine anti-inflammatory treatment is not yet standard for most patients with T2D [9,10,11,56,57,58,59,60,61,62,63,64,65]. Future strategies may require the selection of patients with dominant inflammatory phenotypes, using molecular or translational biomarkers to identify those most likely to benefit.

Overall, contemporary therapies should be viewed as modulators of a molecular network rather than isolated correctors of single risk factors. Their clinical benefit likely derives from simultaneous effects on hemodynamic, metabolic, inflammatory, hepatic, renal, vascular, and fibrotic pathways.

## 9. Future Perspectives

The 2026 CKM guideline provides a clinical counterpart to this biological model by encouraging staged identification and interdisciplinary management of cardiovascular, kidney, and metabolic risk [8]. Future molecular staging could be aligned with this framework by linking dominant pathway signatures, such as adipose-insulin-resistant, liver-lipotoxic, kidney-fibrotic, vascular-oxidative, and inflammatory-thrombotic phenotypes, to staged CKM prevention and treatment algorithms.

Effective management of cardiometabolic residual risk in T2D requires a paradigm shift from isolated risk-factor control to pathway-oriented phenotyping. Conventional targets such as HbA1c, LDL cholesterol, blood pressure, body weight, eGFR, and albuminuria remain essential, but they do not fully describe the biological processes that continue to drive organ damage.

A key challenge will be the identification of dominant residual-risk phenotypes. Some patients may be primarily characterized by an insulin-resistant adipose phenotype, others by a liver-metabolic phenotype, a kidney-vascular phenotype, an inflammatory phenotype, a thrombo-inflammatory phenotype, or mixed patterns. Recognizing these patterns may help guide more individualized prevention strategies.

Omics technologies may contribute to this transition. Proteomics, metabolomics, lipidomics, transcriptomics, epigenomics, and microbiome profiling could identify molecular signatures of residual risk and clarify the pathways linking T2D to cardiovascular, renal, hepatic, and microvascular complications. Candidate molecular layers include lipid species, ceramide profiles, hepatokines, inflammatory mediators, fibrosis markers, endothelial proteins, and circulating markers of renal injury.

Artificial intelligence and machine-learning approaches may support integration of clinical data, laboratory parameters, imaging, digital health information, and multi-omics biomarkers. Their greatest value would not be simple risk prediction, but biological deconvolution: identifying whether persistent risk is mainly driven by adipose dysfunction, hepatic lipotoxicity, renal microvascular injury, endothelial dysfunction, or inflammatory-fibrotic activation.

A pragmatic near-term approach may be the integration of simple clinical indices with selected organ-specific and inflammatory markers. TyG-derived indices, adiposity markers, FIB-4, hepatic steatosis scores, albuminuria, eGFR, lipid-related markers, and inflammatory biomarkers could generate an accessible residual-risk profile while more advanced molecular tools are being validated.

The future goal is therefore not simply to predict who is at higher risk, but to understand why that risk persists. A pathway-centered approach may allow clinicians to match therapeutic strategies to the dominant biological drivers of residual risk and move toward precision prevention in T2D.

## 10. Limitations

This review has several limitations. First, it is a narrative rather than a systematic review; therefore, the literature selection was designed to provide a clinically and mechanistically coherent synthesis rather than a formally exhaustive or PRISMA-based evidence map. Second, several biomarkers and composite indices discussed in the manuscript, including TyG-derived indices, VAI, LAP, AIP, HSI, FIB-4, albuminuria, and eGFR, are indirect clinical proxies rather than direct molecular measurements of intracellular kinase activation, inflammasome assembly, mitochondrial function, eNOS coupling, hepatocyte lipotoxicity, or podocyte stress.

Third, pathway-centered phenotyping remains an emerging strategy and should not yet be interpreted as a fully validated clinical decision algorithm. Some mechanistic links, particularly those involving AMPK/SIRT1/PGC-1α signaling, sodium-hydrogen exchanger modulation, hepatokine signaling, inflammasome activity, and selected endothelial pathways, are supported mainly by experimental and translational evidence. These mechanisms are useful for biological interpretation, but their relative contribution to clinical outcomes may vary across patient phenotypes, therapeutic contexts, and disease stages.

## 11. Conclusions

Cardiometabolic residual risk in T2D is the clinical expression of persistent molecular activity rather than merely incomplete achievement of conventional therapeutic targets. Insulin resistance, stress-kinase activation, lipotoxicity, metaflammation, oxidative stress, mitochondrial dysfunction, MASLD, diabetic kidney disease, endothelial dysfunction, thrombo-inflammation, and fibrosis interact within a systemic network involving adipose tissue, liver, kidney, immune cells, and vasculature.

This network explains why cardiovascular, renal, hepatic, and microvascular complications may continue to develop despite apparently adequate control of individual risk factors. The persistence of risk may reflect ongoing pathway activation, tissue remodeling, and metabolic or epigenetic memory rather than failure of a single clinical target.

Simple translational biomarkers and clinical indices may help bridge molecular complexity and everyday practice. They cannot replace molecular profiling, but they may provide accessible windows into insulin resistance, adipose-lipid dysfunction, hepatic injury, renal-microvascular damage, and vascular inflammation.

Future cardiometabolic care should therefore move from a purely target-centered model toward a pathway-centered model. Identifying the dominant biological drivers of residual risk may allow more precise use of lifestyle, pharmacological, and organ-protective strategies, ultimately improving long-term outcomes in patients with T2D.

## Figures and Tables

**Table 1:** Molecular pathways, clinical proxies, and therapeutic modulation of cardiometabolic residual risk in type 2 diabetes.

Molecular Pathway	Main Organ Axis	Key Molecular Mechanisms	Clinical/Biochemical Proxies	Potential Therapeutic Modulation
Insulin resistance and metabolic stress	Adipose tissue, skeletal muscle, liver, endothelium	Impaired insulin receptor substrate and PI3K/Akt signaling; compensatory hyperinsulinemia; reduced metabolic flexibility; glucotoxicity and lipotoxicity.	HbA1c, fasting glucose, triglycerides, TyG index, TyG-BMI, waist/BMI, HOMA-IR when available.	Weight reduction, physical activity, nutritional intervention, GLP-1 RA, dual GIP/GLP-1 receptor agonism, SGLT2i.
Adipose dysfunction and metaflammation	Visceral adipose tissue, immune system	Adipocyte hypertrophy, macrophage infiltration, NLRP3 inflammasome activation, IL-1β/IL-6/TNF-α signaling, altered adipokines.	BMI, waist circumference, VAI, LAP, hs-CRP or inflammatory markers when available.	Weight loss, exercise, incretin-based therapies, improvement of visceral adiposity, possible future anti-inflammatory strategies.
Oxidative and mitochondrial stress	Liver, kidney, vasculature, skeletal muscle, beta cells	Excess reactive oxygen species, mitochondrial dysfunction, impaired mitophagy, endoplasmic reticulum stress, AGE-RAGE activation.	Indirectly reflected by hyperglycemia, albuminuria, endothelial dysfunction, MASLD markers; no single routine marker.	Glycemic optimization, lifestyle, SGLT2i, GLP-1 RA, reduction in lipotoxic and inflammatory burden.
MASLD and hepatic metabolic dysfunction	Liver–adipose–vascular axis	Selective hepatic insulin resistance, de novo lipogenesis, ceramides/diacylglycerols, hepatokines, stellate cell activation, fibrosis.	ALT/AST/GGT, HSI, fatty liver index, FIB-4, imaging or elastography when available.	Weight loss, GLP-1 RA, dual GIP/GLP-1 receptor agonism, metabolic risk-factor control, emerging liver-directed strategies.
Diabetic kidney disease and renal inflammation	Kidney–heart–vascular axis	Glomerular hyperfiltration, podocyte injury, tubular hypoxia, RAAS activation, mineralocorticoid receptor activation, inflammation and fibrosis.	eGFR, albuminuria, albumin-to-creatinine ratio, blood pressure, potassium monitoring when appropriate.	RAAS blockade, SGLT2i, finerenone, blood pressure control, glycemic and metabolic optimization.
Endothelial dysfunction	Macrovascular and microvascular beds	Reduced nitric oxide bioavailability, impaired PI3K/Akt signaling, oxidative stress, leukocyte adhesion, endothelial permeability.	Albuminuria, blood pressure, pulse pressure, vascular complications, microvascular disease markers.	Lifestyle, lipid lowering, antihypertensive therapy, SGLT2i, GLP-1 RA, smoking cessation.
Atherogenic dyslipidemia	Liver-vascular axis	Triglyceride-rich lipoproteins, remnant cholesterol, low HDL cholesterol, small dense LDL, lipid oxidation and plaque progression.	LDL-C, non-HDL-C, triglycerides, HDL-C, AIP, apoB when available.	Statins, ezetimibe, PCSK9-targeted therapy, triglyceride-focused strategies in selected patients, weight loss.
Thrombo-inflammation	Vasculature, platelets, immune cells	Platelet hyperreactivity, endothelial activation, tissue factor expression, impaired fibrinolysis, inflammatory leukocyte recruitment.	History of ASCVD, inflammatory markers, platelet-related risk context; no simple routine composite marker.	Aggressive risk-factor control, antiplatelet therapy when indicated, lipid lowering, weight reduction, future phenotype-guided anti-inflammatory approaches.
Fibrosis and tissue remodeling	Liver, kidney, heart, vasculature	TGF-β signaling, extracellular matrix deposition, stellate cell activation, mineralocorticoid receptor signaling, chronic wound-healing response.	FIB-4, elastography/imaging when available, albuminuria, eGFR trajectory, heart failure phenotype.	Finerenone for DKD, RAAS blockade, SGLT2i, incretin-based therapies for metabolic liver disease, emerging anti-fibrotic strategies.
Integrated residual-risk phenotype	Adipose–liver–kidney–vascular network	Persistence of multiple active pathways despite apparent control of individual clinical targets.	Combined profile: TyG/TyG-BMI, VAI/LAP, AIP, HSI/FIB-4, albuminuria/eGFR, inflammatory markers.	Pathway-centered care integrating lifestyle, lipid lowering, kidney protection, incretin-based treatment, SGLT2i, finerenone, and individualized risk management.

## Data Availability

No new data were created or analyzed in this study. Data sharing is not applicable to this article.

## Abbreviations

The following abbreviations are used in this manuscript:

ACC, American College of Cardiology; ADA, American Diabetes Association; AGE, advanced glycation end-product; AHA, American Heart Association; AIP, atherogenic index of plasma; ALT, alanine aminotransferase; AMPK, AMP-activated protein kinase; ASN, American Society of Nephrology; ASC, apoptosis-associated speck-like protein containing a CARD; ASCVD, atherosclerotic cardiovascular disease; AST, aspartate aminotransferase; BH2, dihydrobiopterin; BH4, tetrahydrobiopterin; BMI, body mass index; ChREBP, carbohydrate-responsive element-binding protein; CKD, chronic kidney disease; CKM, cardiovascular-kidney-metabolic; DAG, diacylglycerol; DKD, diabetic kidney disease; eGFR, estimated glomerular filtration rate; eNOS, endothelial nitric oxide synthase; FGF21, fibroblast growth factor 21; FIB-4, fibrosis-4 index; FoxO1, forkhead box O1; GGT, gamma-glutamyl transferase; GIP, glucose-dependent insulinotropic polypeptide; GLP-1 RA, glucagon-like peptide-1 receptor agonist; GLUT4, glucose transporter type 4; HbA1c, glycated haemoglobin; HDL-C, high-density lipoprotein cholesterol; HOMA-IR, homeostatic model assessment of insulin resistance; HSI, hepatic steatosis index; hs-CRP, high-sensitivity C-reactive protein; IKKβ, I-kappa-B kinase β; IL, interleukin; IRS, insulin receptor substrate; JNK, c-Jun N-terminal kinase; LAP, lipid accumulation product; LDL-C, low-density lipoprotein cholesterol; MAPK, mitogen-activated protein kinase; MASLD, metabolic dysfunction-associated steatotic liver disease; MASH, metabolic dysfunction-associated steatohepatitis; NADPH, nicotinamide adenine dinucleotide phosphate; NF-kappaB, nuclear factor kappa B; NLRP3, NLR pyrin domain-containing 3; oxLipids, oxidized lipids; PGC-1α, peroxisome proliferator-activated receptor gamma coactivator 1-alpha; PI3K, phosphoinositide 3-kinase; PKC, protein kinase C; PKCε, protein kinase C epsilon; PP2A, protein phosphatase 2A; RAAS, renin-angiotensin-aldosterone system; RAGE, receptor for advanced glycation end-products; SGLT2i, sodium-glucose cotransporter 2 inhibitor; SIRT1, sirtuin 1; SREBP-1c, sterol regulatory element-binding protein 1c; T2D, type 2 diabetes mellitus; TGF-β, transforming growth factor beta; TLR4, toll-like receptor 4; TNF-α, tumour necrosis factor alpha; TRPC6, transient receptor potential cation channel C6; TyG, triglyceride-glucose index; VAI, visceral adiposity index; VLDL, very-low-density lipoprotein.
